# Reverse total shoulder arthroplasty is a surgical option for patients over 60 years of age with locked shoulder dislocation

**DOI:** 10.1007/s00402-026-06358-5

**Published:** 2026-06-06

**Authors:** Emilio Sebastia-Forcada, Francisco Antonio Miralles-Muñoz, Daniel Martinez-Mendez, Luis Albero-Catala, Vicente Climent-Peris, Alejandro Lizaur-Utrilla

**Affiliations:** 1 Hospital General de Elda, Alicante, Spain; 2 Miguel Hernandez University, Elche, Spain

**Keywords:** Locked, Shoulder, Dislocation, Arthroplasty, Constant score

## Abstract

**Background:**

in locked dislocation of the shoulder, instead of reducing back to the glenoid, the humeral head remains incarcerated on the glenoid in a locked fashion. This clinical situation is fairly uncommon. It is essential to conduct an individual evaluation of each patient to determine the appropriate treatment.

**Objective:**

the aim of this study was to evaluate the functional outcomes of reverse total shoulder arthroplasty (rTSA) in the treatment of locked shoulder dislocation.

**Methods:**

patients with locked shoulder dislocation who underwent reverse shoulder prosthesis surgery and were admitted to our center between 2007 and 2023 were reviewed. The primary outcome was the Constant score. Secondary outcomes included the adjusted Constant, UCLA and DASH scores. Additionally, any signs of radiologic loosening were also documented.

**Results:**

the series consisted of 10 patients, six men and four women, with a mean age of 68.0 years. The average time from the traumatic injury to surgery was 7.5 months. All patients showed improved Constant, Adapted Constant, UCLA, and DASH scores compared to their preoperative values. When comparing the outcomes of chronic posterior and anterior dislocations, no differences in functional outcomes or shoulder motion were observed after rTSA implantation. There were no complications during or after surgery.

**Conclusion:**

The results of the present study have shown that patients with locked shoulder dislocation can achieve reliable short-term functional results when treated with rTSA. This proceduredecreases pain, improves functionality and enhances patient satisfaction. Level evidence: IV.

## Introduction

Neglected shoulder dislocation is a rare clinical condition that occurs when an acute dislocation goes undiagnosed. In most cases, the diagnosis is only made once the injury has become chronic and the shoulder has locked up, often due to the retraction and fibrosis of the capsule and soft tissuesas well as the presence of significant bone defects in the glenoid cavity and the humeral head. Therefore, locked shoulder dislocation is a consequence of a delayed diagnosis, making joint reduction impossible through non-invasive methods [4].

Various therapeutic options have been proposed, ranging from conservative treatments for elderly patients with low functional demands [4] to more invasive procedures such as open reduction and preservation of humeral head [4], the McLaughlin procedure [7], allograft reconstruction [6], osteotomy [22] and shoulder arthroplasty [17, 18]. In the context of prosthetic surgery, several authors have proposed different types of treatment: hemiarthroplasty with autograft glenoid reconstruction [18], total shoulder arthroplasty [9, 12, 21] and reverse total shoulder arthroplasty (rTSA) [13, 17].

There are few publications available on rTSA for locked shoulder dislocation [2, 8, 13, 17]. Some authors focused their research on neglected cases [2] or locked anterior shoulder dislocation [8, 17], while others conducted multicenter studies using different types of prostheses [13]. Furthermore, high rates of recurrent instability and other complications have been reported [9].

The purpose of this study was to investigate the clinical and radiographic outcomes of patients with a locked shoulder dislocation treated with rTSA with a minimum postoperative follow-up of 2 years.

## Materials and methods

A retrospective study was conducted on patients who had neglected shoulder dislocations that progressed to a locked dislocation. Neglected dislocation was defined as lasting more than three weeks [2]. All patients admitted to our center between 2007 and 2023 with a locked shoulder dislocation were reviewed. Our institution’s Clinical Research Ethics Committee approved the study.

**Fig. 1:**
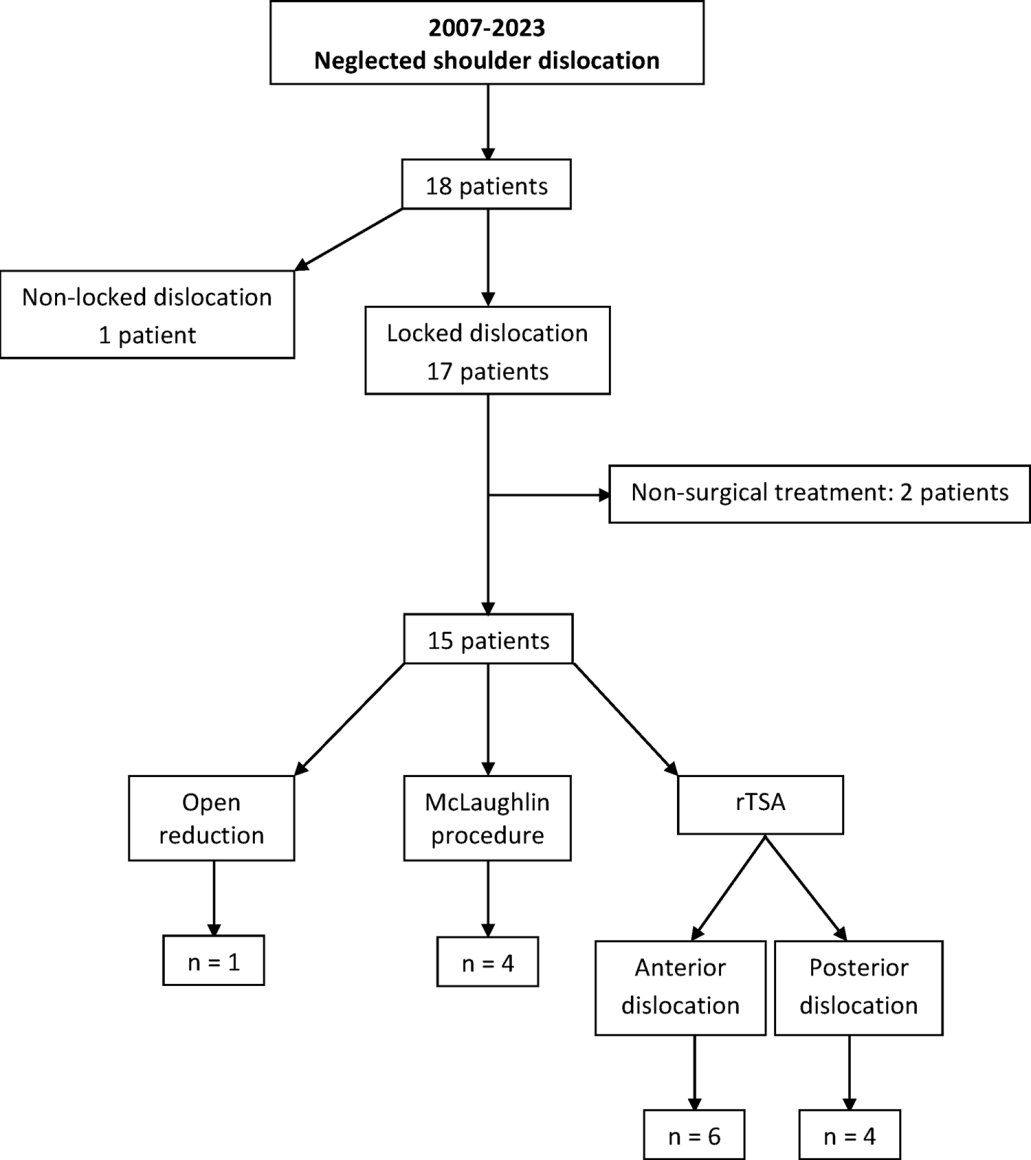
Flowchart of our patients in the study

The inclusion criteria for this study were anterior or posterior locked dislocation of the shoulder joint treated with rTSA, complete clinical and radiographic data available before surgery and at final follow-up and a minimum of 2 years of follow-up. All cases managed with a technique different from rTSA were excluded from the study. Closed reduction was achieved in one patient with a shoulder dislocation of 25 days’ duration, while another patient with a shoulder dislocation of six weeks’ duration required open reduction. Surgical treatment was not considered for two elderly patients with locked anterior shoulder dislocations, and four young patients with locked posterior shoulder dislocations underwent the McLaughlin procedure (Fig. 1).

Therefore, between 2007 and 2023, ten patients with locked shoulder dislocation were treated in our department using rTSA. Six men and four women, with an average age of 68.0 years (range: 60–76 years) formed the sample for this study. There were six cases of locked anterior dislocations and four cases of locked posterior dislocations. The average time from traumatic injury to surgery was 7.5 months (range: 3–21 months).

The delay in surgical treatment was caused by several reasons. In the four patients with posterior dislocation, a diagnostic error was made by failing to identify the injury on the initial radiographs following the acute trauma. In the cases of locked anterior shoulder dislocation, three patients were admitted with multiple fractures following a traffic accident, and priority was given to treating life-threatening injuries. Meanwhile, three other patients, with cognitive deficits, were not transferred to a healthcare for performing diagnostic tests after trauma.

All patients underwent clinical and radiological evaluations before surgery and at 3, 6, 12 and 24 months postoperatively. At our center, all clinical and radiological data from each consultation before and after surgery are systematically and routinely entered into a database. Therefore, this review is based on prospectively collected data.

The main variable was the Constant score. The secondary variables were the adjusted Constant, UCLA (University of California-Los Angeles) and Quick-DASH (Disabilities of the Arm, Shoulder and Hand) scores. Additionally, pain was assessed using the visual analog scale (VAS) from 0 to 10 points, where 0 represented no pain, while 10 indicated severe pain. Range of motion in flexion and abduction was measured using a goniometer, and rotations were evaluated based on hand position according to the Constant and Murley questionnaire [3]. Although patient satisfaction is included in the UCLA score with only 2 possible responses (yes/no), in all the evaluations, patient satisfaction with the final result is assessed using a VAS ranging from 0 (very dissatisfied) to 10 (very satisfied).

**Fig. 2:**
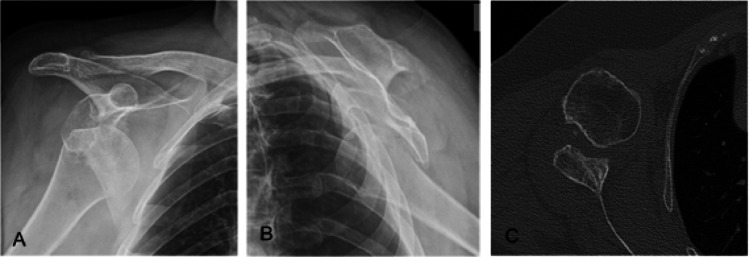
**A** and **B** AP and transscapular X-rays view. **C **CT scan showing chronic shoulder dislocation with anteroinferior bone defect

 A radiological evaluation of the shoulder was performed using anteroposterior and transscapular views. Signs of loosening of the stem and glenoid were documented based on the radiographic zones established by Melis et al. [10]. Additionally, the scapular notch was assessed using the Sirveaux classification [15]. In all cases, a CT scan was done before surgery for good preoperative planning (Fig. 2), to assess bone defects in the humeral head and glenoid. No ultrasounds or MRIs were used to evaluate the integrity of the rotator cuff. During surgery, the severity of the glenoid defect was assessed using the classification system developed by Antuna et al. [1].

Postoperative complications were recorded, with instability, infection, loosening, and periprosthetic fracture considered relevant. Infection was confirmed by the presence of a productive fistula with positive cultures. Secondary surgeries and their causes were also documented.

The data collected during the final evaluation at 2 years were chosen for the analysis of the results.

### Surgical procedure

**Fig. 3:**
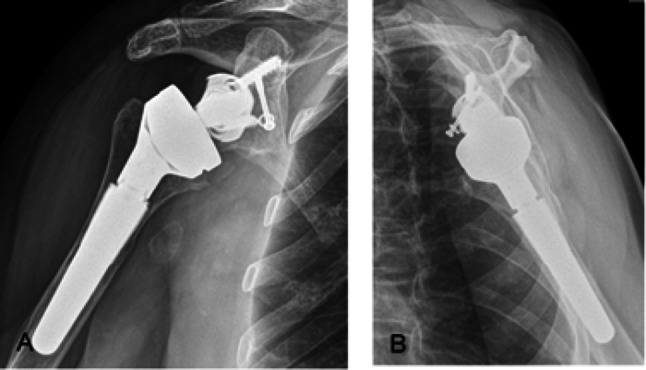
**A** and **B** X-ray at 4 years of assessment

All patients were operated on by the head of the shoulder unit (ESF). With the patient in a beach-chair position, a deltopectoral approach was used. The SMR type reverse total modular shoulder prosthesis (Lima Corporate, Udine, Italy) was implanted in all patients included in the study (Fig. 3).

**Fig. 4:**
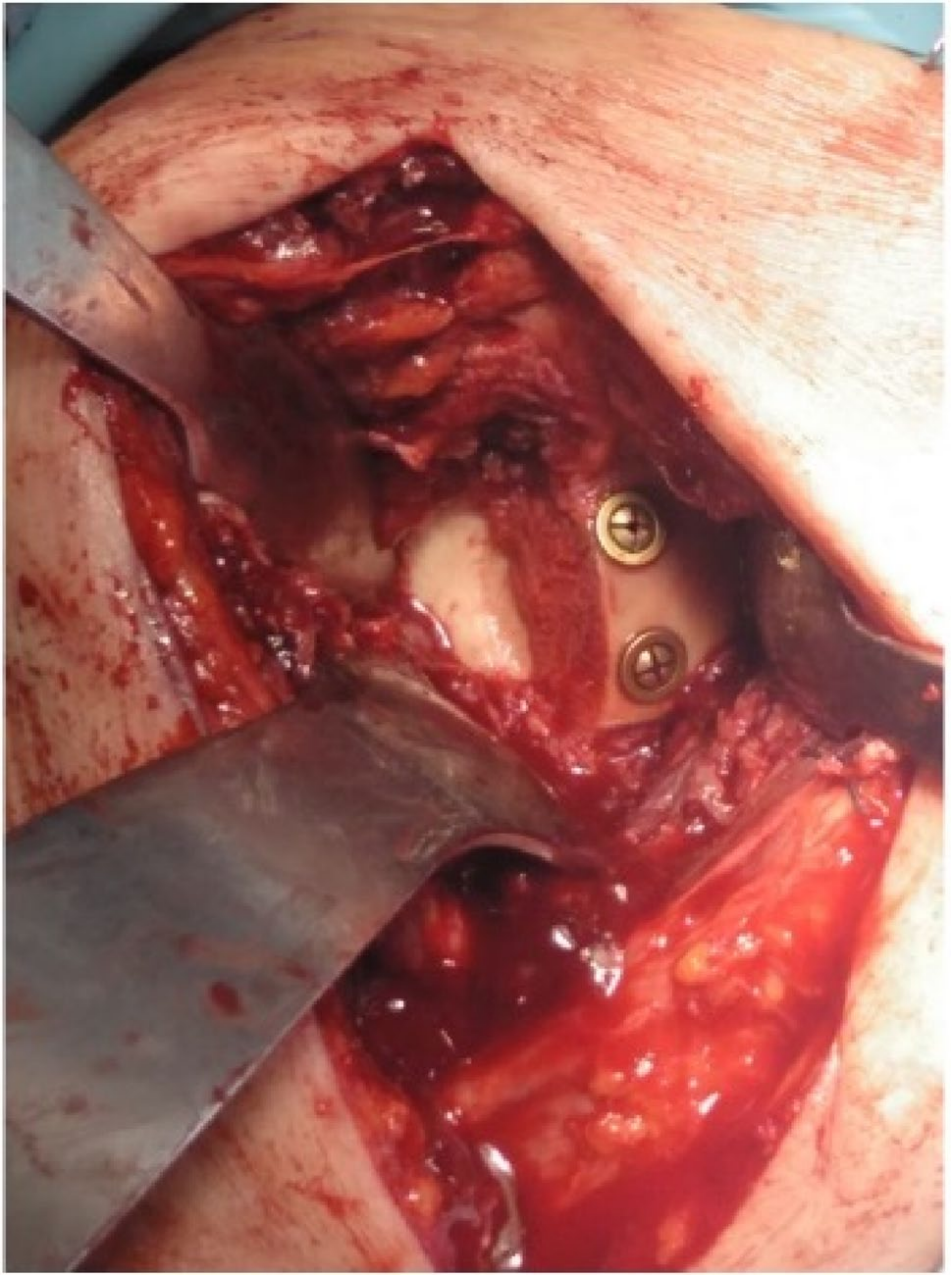
Patient in beach-chair position, with deltopectoral approach. Surgical image with anterior bone graft

Tenotomy with tenodesis of the long head of the biceps and release of the subscapularis tendon with subsequent transosseous suture were performed. After osteotomy of the humeral head, the posterior capsule was tightened and sutured to the posterior rim of the glenoid fossa and the subscapularis tendon was sutured in neutral rotation following the release of the muscular area of the scapula. These surgical procedures, which involve the capsular and tendon structures, are performed for both posterior and anterior dislocations. Degenerative lesions of the supraspinatus tendon that were detected intraoperatively were not repaired. The humeral head was used as a bone graft in cases of glenoid bone defects when the surrounding bone was unable to adequately support the central pin. The size of the graft was adjusted to fit the defect and fixed with cancellous screws (Fig. 4). The size of the sphere was consistent in all instances. The humeral stem was positioned at a 20-degree retroversion angle.

Passive-assisted physiotherapy started the week after the surgery, with the shoulder immobilized for 3 weeks.

### Statistical analysis

Data were analyzed using IBM SPSS version 25 software (IBM Corp, Armonk, NY, USA). Wilcoxon signed rank tests were performed to compare preoperative and postoperative changes in range of motion and clinical outcome scores. For all statistical tests, P < 0.05 was used to determine significance.

## Results

The mean Constant-Murley score significantly increased from 20.5 points preoperatively to 57.5 points postoperatively (*p* < 0.001). The adjusted Constant, UCLA, and Quick-DASH scores all showed significant increases after RSTA compared to their preoperative states (*p* < 0.001). Shoulder abduction increased from 52° before the operation to 111° after, while shoulder flexion improved from 73° pre-arthroplasty to 124° post-arthroplasty (*p* < 0.001). Regarding shoulder rotations, both external (*p* < 0.001) and internal (*p* = 0.022) improved significantly after prosthetic surgery. Patient satisfaction significantly improved after surgical treatment (*p* < 0.001). Detailed information can be found in Table 1.

**Table 1:** Outcome variables

Variable	Before rTSA	After rTSA^1^	*p*
Constant score (points)	20.5 (2.9)	57.5 (2.8)	0.000
Adjusted constant score (points)	27.2 (4.3)	78.0 (8.1)	0.000
UCLA score (points)	10.0 (1.6)	29.6 (1.2)	0.000
Quick-DASH score (points)	63.1 (9.6)	11.7 (2.2)	0.000
VAS-pain	7.1 (1.5)	0.8 (1.1)	0.000
Abduction(º)	52.0 (15.4)	111.0 (19.6)	0.000
Forward flexion(º)	73.0 (16.3)	124.0 (21.7)	0.001
External rotation^2^	1.9 (0.5)	4.3 (0.6)	0.000
Internal rotation^2^	1.6 (0.9)	2.9 (0.7)	0.022
VAS-satisfaction	0.9 (0.8)	7.8 (1.0)	0.000

^1^Results at 2 years of postoperative follow-up

^2^Scores according to the Constant scale

During the 2-year postoperative follow-up, none of the patients showed any periprosthetic radiological signs, and there were no observed cases of loosening of the glenoid or humeral component.

During surgery, complete supraspinatus tendon tears were detected in five patients: four with anterior dislocations and one with a posterior dislocation. However, these injuries were not repaired during the surgical procedure. In the remaining cases, signs of tendon degeneration were observed, but no tears were present.

Two patients required anterior bone grafting for an anterior glenoid defect caused by an anterior marginal fracture, which could potentially compromise the stability of the glenoid component. Although preoperative planning always involved analyzing the percentage of bone loss on CT scans, the final decision was made intraoperatively by the surgeon after evaluating the bone stock. In these cases, autologous bone graft from the resected humeral head was used to enhance the glenoid bone stock before inserting the metaglenoid of the rTSA. Three more patients had minor glenoid defects that did not require bone grafting, as the fixation and stability of the glenoid component were not compromised (Table 2). All patients presented with impact bone lesions on the humeral head. In non-prosthetic surgery, their impact on outcomes is clear. However, in prosthetic replacement, these humeral head deformities do not have predictive value for outcomes, unlike glenoid bone defects.

**Table 2:** Distribution of preoperative bone loss on glenoid according to classification of Antuna et al. [1]

	Mild	Moderate	Severe
Central	-	-	-
Peripheral	1	-	-
Combinate	2	2	-

No intraoperative complications were recorded and there were no cases of postoperative instability, infection or neuropathies.

## Discussion

The results of the present study have shown that patients with locked shoulder dislocation can achieve reliable short-term functional results when treated with rTSA. All patients experienced functional improvement, with reduced pain and increased satisfaction with the outcome, two years after the surgical procedure. Furthermore, no postoperative instabilities were recorded. But it is true that the limitations inherent in this case series require us to take all these statements about the results obtained with caution. Therefore, we consider it important to place them in context.

Shoulder arthroplasty is recommended when the bony injury affects more than 20–25% of the glenoid and 40% of the humeral head articular surface [21]. The rTSA is regarded as an effective treatment for locked shoulder dislocations in older patients who have significant rotator cuff injuries [16] and provides the advantage of using constrained implants to address ongoing instability [14]. A recent systematic review and meta-analysis focused on locked posterior dislocations revealed that anatomical total shoulder arthroplasty was linked to a reduced rate of postoperative complications and revision surgeries compared to shoulder hemiarthroplasty [4]. Matsoukis et al. [9] concluded that shoulder arthroplasty in patients with a fixed anterior shoulder dislocation is challenging and can lead to complications. Although arthroplasty effectively reduces shoulder pain, we agree with the authors who consider it important to explain to the patient the likely limited functional outcomes [9].

Statz et al. [17] found that instability was significantly more common after anatomic arthroplasty than reverse arthroplasty for chronic anterior dislocation. In line with the results of the present study, Van Tongel et al. [19] also reported no surgical complications in their case series, leading them to determine that rTSA offers positive results for chronic glenohumeral dislocation cases. Conversely, Raiss et al. [13] observed that although patients experienced significant functional improvements following rTSA, the average Constant score did not reflect a meaningful clinical benefit across the cohort. Furthermore, their study highlighted a notably high rate of complications post-surgery, which frequently necessitated secondary surgical interventions.In a study by Sahu et al. [14]., it was reported that instability emerges as the most frequent complication when treating locked shoulder dislocations with an anatomical prosthesis. Conversely, the same study found no instances of dislocation following rTSA treatment.

Central to the success of rTSA is the precise and careful management of the glenoid. This assessment guides decisions on reaming techniques, baseplate positioning, and the use of bone grafts when necessary. Some authors [20] recommend placing baseplates in at least 50% of the native bone, using longer central pins when the glenoid defect is greater than 30%, or opting for a screw-retained bone graft when it exceeds 40%. A glenoid bone stock of less than 50% may be crucial to these implants’ survival, according to Formaini et al. [5]. An iliac crest transplant may be helpful for repair when there is a 60–80% glenoid deficit. Following the consolidation of the bone graft, a two-stage reconstruction is advised if the glenoid implant lacks primary stability [14].

According to the research conducted by Wooten et al. [21], a critical observation has been made regarding the temporal relationship between injury and surgical treatment. The study reveals that patients who underwent surgery within one year of experiencing a dislocation exhibited significantly better functional outcomes compared to those who had their surgery more than a year after the injury. However, the review study by Smoak et al. [16] found that delaying surgery did not have a negative impact on the outcomes.

When comparing posterior and anterior locked shoulder dislocations, we have not found significant differences in functional outcomes or mobility following rTSA implantation. Raiss et al. [13] also found no significant differences in outcomes between patients with anterior or posterior dislocations. Several publications have shown that the subscapularis muscle contraction or repair following implantation does not result in instability because of the semi-constrained nature of the rTSA prosthesis [11, 17]. They found that when using a total anatomical prosthesis, problems with the subscapularis muscle could lead to higher anterior instability following surgery [11, 17].

The strength of the present study mainly lies in the fact that all surgeries were performed by the same surgeon with extensive experience in shoulder prosthesis surgery and the same type of prosthesis was used in all cases of the series. But this study has some limitations. The results of the rTSA have not been compared with a control group. The retrospective design and small sample size are inherent characteristics of case series, primarily due to the low incidence of the disease studied. Therefore, the interpretation of results should always be approached with caution in case series studies with small samples.In this scenario, data analysis is limited to being descriptive. The study does not have sufficient statistical power to make comparisons between subgroups.

## Data Availability

No datasets were generated or analysed during the current study.
